# In this month’s *Bulletin*

**DOI:** 10.2471/BLT.16.000716

**Published:** 2016-07-01

In the editorial section David Clarke et al. (482) explain that many countries need to revise their laws to support universal health coverage. Gretchen A Stevens et al. (483) introduce new reporting guidelines for health estimates. Rosanna Baker et al. (484) provide young people with ways to promote understanding of disease risks. 

 Patralekha Chatterjee (487–488) reports on attempts made in India to improve air quality.  Fiona Fleck (489–490) interviews Jeffrey D Sachs on the importance of accounting for the health impacts of energy policies.

## Algeria, Cameroon, Côte d’Ivoire, Ethiopia, Ghana, Guinea, Kenya, Malawi, Mozambique, Nigeria, Rwanda, South Africa, Uganda, United Republic of Tanzania

## Cars versus people

Davies Adeloye et al. (510–521) count the burden of road traffic crashes, injuries and deaths.

## Kenya

## Urban slums and chronic diseases

Samuel Oji Oti et al. (501–509) study hypertension diagnosis and treatment in the community.

## Guinea, Liberia, Sierra Leone

Henry B Perry et al. (551–553) use the Ebola response to argue that community health workers can help build resilient health systems.

## Global

## Not enough research?

Tara M Tancred et al. (491–500) recommend training in health policy and systems research.

## Detecting schistosomes

Anthony Danso-Appiah et al. (522–533) review the evidence for accuracy of a diagnostic test.

## Complying with the *International Health Regulations*

Géraldine Marks-Sultan et al. (534–539) make a case for creating national public health laws where none exist.

## Selling salt, fat and sugar

Vivica I Kraak et al. (540–548) tally the progress made achieved in restricting marketing to children.

## Building research capacity

Rita Kabra et al. (549–550) report on a new approach from the programme on human reproduction. 

## Where there is no radiologist

Aaron L Berkowitz (554–556) calls for treatment protocols in situations where ischaemic and haemorrhagic strokes cannot be reliably differentiated. 

